# Learning Theories Versus Practice: How Do Internal Medicine Residents Study for Licensing Examinations?

**DOI:** 10.7759/cureus.50052

**Published:** 2023-12-06

**Authors:** Priyanka Majety, Yazan Daaboul, Joseph Rencic

**Affiliations:** 1 Endocrinology, Diabetes and Metabolism, Beth Israel Deaconess Medical Center, Harvard Medical School, Boston, USA; 2 Cardiology, Baim Clinical Research Institute, Boston, USA; 3 Internal Medicine, Boston Medical Center, Boston, USA

**Keywords:** exam preparation, abim exam, resident education, adult learning theories, study habits, internal medicine residents

## Abstract

Objective: The ability to recall relevant medical knowledge within clinical contexts is a critical aspect of effective and efficient patient diagnosis and management. The ever-growing and changing body of medical literature requires learners to develop effective life-long learning techniques. Learners can more successfully build their fund of knowledge and ability to retrieve it by using evidence-based learning strategies. Our objective was to evaluate the study habits of internal medicine (IM) residents at an academic institution to understand if they apply key learning strategies for the American Board of Internal Medicine (ABIM) exam preparation. We also briefly review various learning strategies that can be applied to IM residency curricula.

Methods: A web-based survey consisting of 16 multiple-response questions on study habits was filled out by the IM residents in 2019 at Tufts Medical Center.

Results: Of the 75 residents invited to participate in the study, 69 responded (response rate = 92%). Of the responders, n=25 (36.2%) were post-graduate year (PGY)-1, n=20 (29.0%) were PGY-2, and n=24 (34.8%) were PGY-3 residents. More than half the residents (n=40, 58%) had Step 2 Clinical Knowledge (CK) scores > 250. Residents self-reported applying spaced learning (67%), interleaving (64%), retrieval (64%), and elaboration practices (46%) for exam preparation. There was a significant association between the Step 2 CK score and elaboration (p=0.017) technique but not with spaced learning, interleaving, or retrieval. The majority of residents felt not at all prepared (n=42, 60.9%) for the ABIM exam.

Conclusions: Despite two years of clinical training, 33% of the third-year residents felt inadequately prepared for the board certification exam. Incorporating evidence-based learning strategies into their daily curriculum may help them better prepare for the ABIM exam.

## Introduction

Due to patient care demands, internal medicine (IM) residents spend a significant amount of time providing direct or indirect patient care and have only a limited amount of time to spare for educational activities [1,2]. Specifically, Leafloor et al. highlighted that IM residents spend an average of 41.8% of their time on patient care activities, compared to an average of 13.8% of their time spent on educational activities [3]. For medical trainees, effective and efficient diagnosis and management requires a readily retrievable, broad fund of knowledge. Residents spend a significant amount of time engaging in self-directed learning during training [4]. Studies have shown that learning in medical education does not typically ensure long-term retention of knowledge [5-7].

Many IM residency programs do not have curricula focused on evidence-based learning strategies during training that can lead to successful life-long learning. Understanding and analyzing the study habits of residents may promote curricular changes regarding learning strategies. This, in turn, could help improve residents’ learning, performance on high-stakes examinations, and most importantly, diagnostic and management skills in the long term.

Specific cognitive strategies that have received robust support from decades of research are spaced learning, interleaving, retrieval practice, and elaboration. Several studies suggest that learning is better when two or more exposures to information are separated in time [8,9]. The learning advantage of information that is repeated in a “spaced” fashion is commonly referred to as the spacing effect [10]. The same amount of repeated studying of the same information spaced out over time will lead to greater retention of that information in the long run.

Interleaving is the process of mixing multiple topics, whereas blocking involves practicing one topic at a time before the next. Interleaving improves learning by helping to better distinguish between different types of problems. It also strengthens the association between each kind of problem [11-13]. The two concepts of spacing and interleaving are similar, but essentially spacing is revisiting the study material periodically throughout the course, whereas interleaving is switching between ideas while studying.

Testing is a powerful tool to help with the enhanced retrieval of information [14]. While testing is most often used in educational settings for assessment, another benefit of tests is that they improve memory of the tested information. Repeated practice in recalling information in studying and completing a test result in greater long-term memory retention gains. This phenomenon is known as the testing effect [15].

Elaboration involves connecting new information to pre-existing knowledge. Relating material to prior knowledge or past experiences allows us to develop a deeper understanding of the information [16].

We evaluated the study habits of IM residents at an academic institution to understand if they apply key learning strategies for the American Board of Internal Medicine (ABIM) exam preparation.

This article was previously presented as a meeting abstract at the 2020 SGIM (Society of General Internal Medicine) Annual Meeting on May 7, 2020, in Alabama, USA. 

## Materials and methods

We conducted survey-based research to understand the study habits of IM residents. In November 2019, the web-based survey was emailed to the IM residents at Tufts Medical Center. The survey was developed by the authors through an iterative process and consisted of 16 multiple-response questions on demographics, perceived level of preparedness, confidence in test taking, resources used to study, study session duration, application of spaced learning, interleaving, elaboration, and retrieval practices (see Appendices). The survey items were developed to capture the major strategies that IM trainees use in studying for the ABIM examination based on the author’s experiences. We piloted the survey with 3 chief residents and using their feedback on the items created a final survey after two iterations. The authors used consensus to classify questions as representative of specific evidence-based learning strategies. All the IM residents were eligible and were invited to participate in the survey. They filled out this survey anonymously to determine their study habits for the ABIM exam. No individual subject identifiers (such as name or initials) were collected. Subjects were informed that their participation was voluntary. 

The study objective was explained to all participants, and verbal consent was obtained. The study protocol was approved for exemption by the Tufts Health Sciences Institutional Review Board (IRB# 13545) and was in accordance with the Helsinki Declaration and its later amendments.

Statistical analysis

All statistical analyses were performed using Stata® 14 (StataCorp LP, TX, USA). Data was analyzed for all subjects who had answered at least one question. Characteristics of the study participants, including age, sex, and post-graduate year (PGY) level, were evaluated using descriptive statistics. Ordinal variables of interest, such as level of preparedness and confidence, were first recorded as 4 categories according to the Likert scale and were then dichotomized for subsequent analyses. The association between categorical variables was performed using the Chi-square test. The Cochran-Mantel-Haenszel test was performed to evaluate the association between categorical variables when stratification was conducted. All variables that were associated with univariate analysis with a p-value ≤ 0.10, as well as variables of historical interest, were included in multivariate regression models. All p-values were two-sided. A p-value < 0.05 was considered statistically significant.

## Results

A total of 75 IM residents were invited to participate, of whom 69 responded (response rate = 92%). Of the responders, n=33 (48%) identified as males and n=36 (52%) as females. Also, n=25 (36.2%) were PGY-1 residents, n=20 (29.0%) were PGY-2 residents, and n=24 (34.8%) were PGY-3 residents (Table 1). More than half the residents (n=40, 58%) had Step 2 CK scores > 250 (Table 2). None of the residents felt either fully prepared or very well prepared for the ABIM exam. Most residents, i.e., n=49 (71%), considered themselves either extremely, fairly or somewhat confident test takers. The perceived level of confidence did not correlate with the resident’s sex, Step 2 CK score, level of preparedness, or the duration of time spent on examination preparation. 

**Table 1:** Participant characteristics, common resources, frequency of learning techniques used by IM residents for ABIM exam preparation during residency PGY - Post-graduate year, IM - Internal medicine, ABIM - American Board of Internal Medicine *A total of 75 IM residents were invited to participate in the study, of whom 69 responded (response rate = 92%) and were eligible for the final analysis **The % is calculated based on all the subjects who provided data. Subjects with missing data were excluded from the denominator ***2 people did not respond, total N=67 in this analysis

Participant characteristics	n (%)*
PGY-1	25 (36.2%)
PGY-2	20 (29%)
PGY-3	24 (34.8%)
Total	69
Females	36 (52%)
Males	33 (48%)
Learning technique	% (n/N)**
Interleaving	63.8% (44/69)
Blocking	59.4% (41/69)
Spaced learning	66.6% (46/69)
Retrieval	63.7% (44/69)
Elaboration	46.3% (32/69)
Resources	% (n/N)***
Question bank	89.6% (60/67)
Textbook	37.3% (25/67)
Online videos	31.3% (21/67)
Journals	19.4% (13/67)
Did not use resources	10.1% (7/67)

**Table 2:** Frequency of individuals who had high Step 2 CK ≥ 250, as categorized by learning strategy Step 2 CK - Step 2 Clinical Knowledge *There was a significant association between Step 2 CK score and the Elaboration Technique (p=0.017), but not the remaining techniques

Technique	Achievement of Step 2 CK score > 250		p-value
	Students who did not perform technique % (n)	Students who performed technique % (n)	
Interleaving	60.0% (15/25)	56.8% (25/44)	0.80
Blocking	60.7% (17/28)	56.1% (23/41)	0.70
Spaced Learning	53.4% (11/21)	58.7% (27/46)	0.63
Retrieval	52.2% (12/23)	59.1% (26/44)	0.59
Elaboration	42.9% (15/35)	71.9% (23/32)	0.017*

Most residents used question banks as a study resource (n=60/67, 89.6%) followed by textbooks (n=25/67, 37.3%), online videos (21/65, n=31.3%), and medical journals (13/65, 19.4%) (Table 1). Residents self-reported applying spaced learning (66.6%), interleaving (63.8%), retrieval (63.7%), and elaboration practices (46.3%) for exam preparation (Table 1). Notably, there was a significant association (p=0.017) between the Step 2 CK score and the elaboration technique. In contrast, there was no significant association between Step 2 CK scores and any other techniques (Table 2).

## Discussion

Residents spend a significant amount of time engaging in self-directed learning during training [4]. Studies have shown that learning in medical education does not typically ensure long-term retention of knowledge [5-7]. There is robust evidence to support spaced learning in medical education, especially with expanding intervals [17-20]. Studies are required to understand the implications of other strategies, especially in resident education. Curricula focused on incorporating key learning strategies into their daily routines may help IM residents better prepare for the ABIM examination and life-long learning. Table 3 summarizes the key learning strategies along with examples pertinent to IM residency curricula [21]. Our aim was to assess if the residents have the necessary tools to practice evidence-based learning techniques. 

**Table 3:** Summary of learning strategies discussed with examples pertinent to IM residency curricula ITE - In-training examination, ACE - Angiotensin-converting enzyme, IM - Internal medicine

Learning strategy	Description	Examples pertinent to residency curricula
Spaced learning	Creating a curriculum that spreads study activities out over time	-Carving out time during educational conferences to discuss what was learned during previous conferences. -Learning points from the previous day/week should be revisited at the beginning of a new workday/week. -Multiple choice questions discussed at a board review conference can be emailed out to the residents at periodic intervals. -Implement cumulative exams and quizzes (not ITEs) and provide immediate feedback.
Interleaving	Switching between topics while studying	-Mixing up questions from different subspecialties, rather than study questions from only one subject at a time. -Teaching conferences should discuss a mix of various subtopics, comparing, and contrasting them (lecture on anti-hypertensive medications, instead of teaching in categories of ACE inhibitors, diuretics, calcium channel blockers, etc., these categories can be intermixed or interleaved). -Teaching rounds can be standardized, encouraging attendings to practice interleaving on a day-to-day basis: while discussing the labs and imaging findings related to a topic, compare/contrast with what these findings would be in different diagnoses. Rotations could be designed in such a way they are interleaved allowing residents to come back to a previously attended rotation and test their knowledge. -Programs can make sets of flashcards available to residents.
Retrieval	Bringing learned information to mind from long-term memory	-Asking learners to identify take-home points or new questions that have arisen during a learning session. -Setting the expectation that learners will be asked to identify two key points at the end of the workday (ambulatory clinic, ward rounds, or a didactic session). -Providing learners with low-stakes quizzes before beginning a new rotation (can help identify weak areas) and at the end of the rotation will engage the learner in retrieval and help reduce test anxiety. This should be followed by feedback with correct and incorrect answers with reasoning.
Elaboration	Asking and explaining why and how things work	-During day-to-day rounds, consider asking residents to explain their findings with ‘why’ and ‘what if’ questions that encourage them to understand physiological concepts. -Hypothetical situations can be created to extend their learning. -Encourage all members of the team to teach.

Although there are some data on IM resident use of learning resources during their training [22], our study adds to the literature by analyzing their specific study strategies, especially, spaced learning, blocking, interleaving, elaboration, and retrieval practice during ABIM exam preparation during residency.

A significant proportion of residents report using evidence-based learning strategies to improve their knowledge retention. Although spaced learning and retrieval practice strategies are often built into question banks, the high percentage of residents reporting the use of interleaving suggests that most have an awareness of this learning strategy. The association of elaboration practice with higher Step 2 CK scores is hypothesis-generating but requires further study to prove a causal relationship.

Our study reveals that despite more than 2 years of residency training, 33% of PGY-3 residents report being “not at all prepared.” This high percentage suggests that some residents believe that clinical training does not adequately prepare them for succeeding on the ABIM certification examination. The fact that some ABIM licensing examination questions tested focus on important but uncommon topics could explain this feeling of unpreparedness. Recent data suggest that among questions on IM-Maintenance of Certification (MOC) examinations, only 69% aligned with conditions seen in general internal medicine practices [23].

Our study has limitations. This study was done at a single academic center in Boston and the number of participants was low. Power determination was not done. The survey tool was developed by the authors, and was not validated at other institutions. The perceived level of preparedness for the ABIM exam may have been confounded by the duration of the gap between the timing of our study and the ABIM exam (eleven months). We recognize that there has been an increase in the number of digital education resources available to trainees including podcasts and Twitter but did not analyze if residents used these for exam preparation.

As residency programs are assessed based on the board examination success rate of their graduates, both by future applicants and by the Accreditation Council for Graduate Medical Education (which requires programs to have at least an 80% certification examination first-time pass rate over a three-year period), program directors must be invested in the board examination success rate of their graduates. Curricula focused on how to incorporate key learning strategies into their daily routines may help IM residents better prepare for the ABIM examination.

Additional studies with a larger number of participants are needed to better understand residents’ study habits and come up with strategies to maximize their learning during training. Longitudinal analyses to assess the effectiveness of teaching residents about key learning strategies are needed. Studies on outcomes such as ABIM or ITE scores and learner satisfaction may help understand the utility of such curricula.

## Conclusions

Our study reveals that despite two years of clinical training, 33% of the third-year residents felt inadequately prepared for the board certification examination. Question banks are the most used resources for the American Board of Internal Medicine (ABIM) exam preparation. A significant proportion of internal medicine residents reported applying learning strategies (spaced learning, interleaving, retrieval, elaboration) during the ABIM exam preparation during their residency training at our institution. Further research with a larger number of participants is required to confirm this finding. Incorporating evidence-based learning strategies into the daily curricula of residents and educating them about these effective techniques, ideally early in residency training, may help them better prepare for the ABIM exam and life-long learning.

## Appendices

**Table 4:** Internal medicine residents’ study habits ABIM - American Board of Internal Medicine, PGY - Post-graduate year, Step 2 CK - Step 2 clinical knowledge, AOA - Alpha Omega Alpha, N/A - Not applicable

Question Number	Question	Responses
Q1	In how many months will you take your ABIM (American Board of Internal Medicine) board exam?	
Q2	Which of the following describes your level of preparation to pass the upcoming ABIM board exam?	Not at all prepared, Slightly prepared, Moderately prepared, Very well prepared, Fully prepared
Q3	Which of the following resources do you use to study? Check all the options that apply.	Question banks, Journals/articles, Textbooks, Online videos, Other (please specify)
Q4	Which of the following best describes the time you take to prepare for the ABIM board exam?	1 week before the exam, 2 weeks before the exam, 1-2 months before the exam, 3-4 months before the exam, 5-6 months before the exam, More than 6 months before the exam
Q5	How many days in a week do you study when you are on service?	0 days a week, 1-2 days a week, 3-4 days a week, Everyday, Other (please specify)
Q6	How many days in a week do you study when you are on elective?	0 days a week, 1-2 days a week, 3-4 days a week, Everyday, Other (please specify)
Q7	Which of the following describes the duration of your typical study session?	0-15 minutes, 16-30 minutes, 31-60 minutes, More than 60 minutes, Other (please specify)
Q8	When you study, which of the following techniques do you use? (Check all that apply)	Highlighting the relevant/important points in a text, Making notes, Reviewing notes, Writing specific learning goals (based on knowledge deficits) to guide your study prior to reading a topic, Mentally rehearsing the learning points, Discussing with another person, Repeating the questions you answered incorrectly after a few days/weeks. Repeating the entire question bank after a few days/weeks, Other (please specify)
Q9	Which of the following describes your approach(es) during your typical study session?	Study one subject per study session, Study multiple subjects per study session, Study one subject in its entirety before moving on to the next subject, Completely random questions/subjects, Other (please specify)
Q10	How often do you read the referenced articles at the end of the answers in a question bank for additional information?	Almost always, Sometimes, Every once in a while, Rarely, Never, Only when I get the answer wrong
Q11	How confident a test-taker are you?	Not at all confident, Slightly confident, Somewhat confident, Fairly confident, Extremely confident
Q12	What is your PGY level?	PGY-1, PGY-2, PGY-3
Q13	What is your gender?	Female, Male, Other
Q14	Are you an international medical graduate?	Yes, No, N/A
Q15	What was your step 2 CK score? (optional)	less than 220, 221-250, greater than 250
Q16	Are you a member of AOA?	Yes, No, N/A
